# Correction: Görte et al. Comparative Proton and Photon Irradiation Combined with Pharmacological Inhibitors in 3D Pancreatic Cancer Cultures. *Cancers* 2020, *12*, 3216

**DOI:** 10.3390/cancers13133364

**Published:** 2021-07-05

**Authors:** Josephine Görte, Elke Beyreuther, Erik H. J. Danen, Nils Cordes

**Affiliations:** 1 OncoRay—National Center for Radiation Research in Oncology, Faculty of Medicine Carl Gustav Carus Technische Universität Dresden, 01307 Dresden, Germany; Josephine.Goerte@uniklinikum-dresden.de (J.G.); Elke.Beyreuther@uniklinikum-dresden.de (E.B.); 2 Institute of Radiooncology—OncoRay, Helmholtz-Zentrum Dresden—Rossendorf, 01328 Dresden, Germany; 3 Institute of Radiation Physics, Helmholtz-Zentrum Dresden—Rossendorf, 01328 Dresden, Germany; 4 Division of Drug Discovery and Safety, Leiden Academic Centre for Drug Research, Leiden University, 2333CC Leiden, The Netherlands; e.danen@lacdr.leidenuniv.nl; 5 German Cancer Consortium, Partner Site Dresden: German Cancer Research Center, 69120 Heidelberg, Germany; 6 Department of Radiotherapy and Radiation Oncology, University Hospital Carl Gustav Carus, Technische Universität Dresden, 01307 Dresden, Germany

The authors wish to make the following corrections to this paper [1]:

In the original article, there was a mistake in Figure 1, Figure 5, Figure S6, Tables S1 and S2 as published [1]. All results involving the cell line Capan-1 were removed. This is due to a recent microsatellite analysis that identified Capan-1 cells to be a different cell line. The manuscript was adapted accordingly, including that in the first sentence of Section 2.1, “six human PDAC cell lines” was changed to “five”; in the second sentence of Section 4.2, “Capan-1 and Colo357 cell lines” was changed to “Colo357 cell lines” only. Another mistake in indicating the cell line names was also corrected in the figure legend of Figure 5 (“Colo357 and MiaPaCa-2” was changed to “indicated PDAC cells”). The corrected Figure 1, Figure 5 and Figure S6 and the corrected Table S1 and Table S2 appear below.

The authors apologize for any inconvenience caused and state that the scientific conclusions remain unaffected. The original article has been updated. 
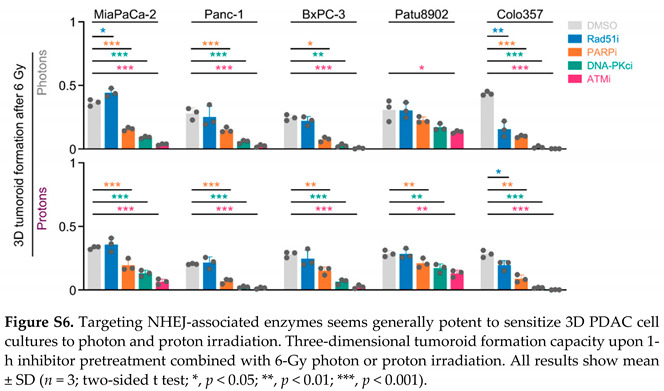
 
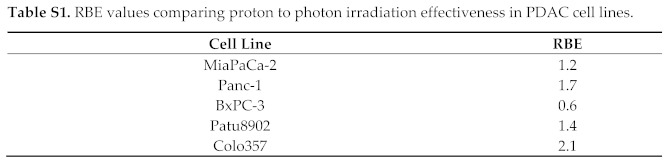
 
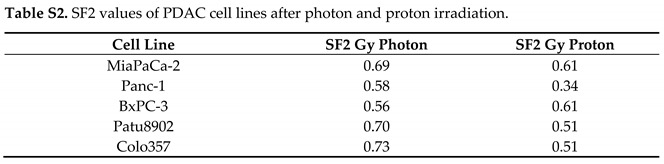


## Figures and Tables

**Figure 1:**
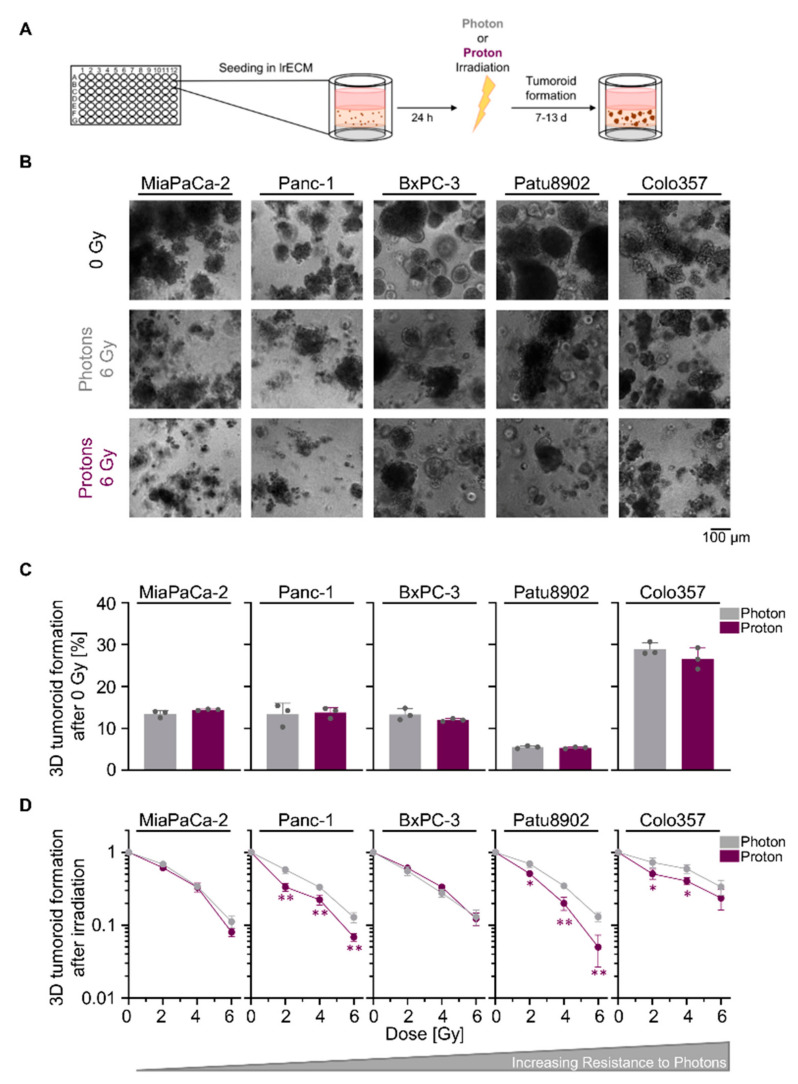
Proton irradiation tends to be more effective in reducing pancreatic ductal adenocarcinoma (PDAC) tumoroid growth than photon irradiation. (**A**) Experimental set-up for examining 3D PDAC tumoroid growth; (**B**) Representative bright-field images of unirradiated, 6-Gy photon and 6-Gy proton irradiated 3D PDAC tumoroids, scale bar: 100 µm; (**C**) 3D tumoroid formation capacity without irradiation; (**D**) 3D PDAC tumoroid growth after irradiation with 2, 4, or 6 Gy of photons and protons. Cell lines are ordered by increasing resistance to photon irradiation. Results show mean ± SD (*n* = 3; two-sided *t*-test; *, *p* < 0.05; **, *p* < 0.01).

**Figure 5:**
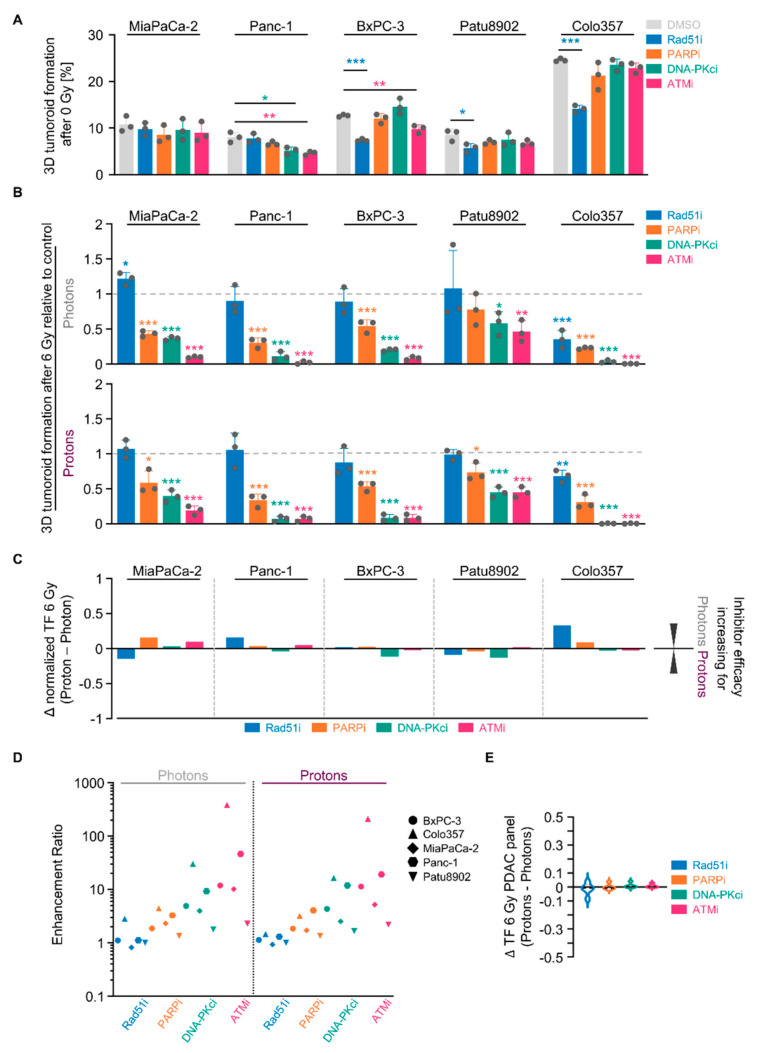
Targeting NHEJ-associated enzymes seems generally potent to sensitize 3D PDAC cell cultures to photon and proton irradiation. (**A**) 3D tumoroid formation capacity of unirradiated indicated PDAC cells treated with indicated DNA repair inhibitors (experimental set-up shown in Figure S2A); (**B**) Normalized 3D PDAC tumoroid formation capacity upon 1-h inhibitor pretreatment combined with 6-Gy photon or proton irradiation; (**C**) Differences in the radiosensitizing efficacy of inhibitors visualized by Δ values of normalized tumor formation capacity (tumoroid formation capacity after 6 Gy of protons - tumoroid formation capacity after 6 Gy of photons); (**D**) Enhancement ratio (tumoroid formation capacity after 6 Gy control treatment/tumoroid formation capacity after 6 Gy inhibitor treatment) of each cell line analyzed in B (see Figure S6) after photon and proton irradiation plotted logarithmically; (**E**) Analysis of differences in the radiosensitizing efficacy of indicated inhibitors showing violin blots of summarized Δ values of tumoroid formation capacity from all PDAC cell lines analyzed in Figure S6. All results show mean ± SD (*n* = 3; two-sided t-test; *, *p* < 0.05; **, *p* < 0.01; ***, *p* < 0.001); Δ: delta, TF: tumoroid formation.
