# Supplementary material for: Comparative Proton and Photon Irradiation Combined with Pharmacological Inhibitors in 3D Pancreatic Cancer Cultures
Source: Cancers (Basel). 2020 Oct 31;12(11):3216. doi: 10.3390/cancers12113216 (PMC7692858; doi:10.3390/cancers12113216)
Supplement: Supplementary file 1 [file cancers-12-03216-s001.zip › cancers-978077-supplementary.pdf]

Supplementary Materials

Comparative proton and photon irradiation combined with pharmacological inhibitors in 3D pancreatic cancer cultures

Josephine Görte, Elke Beyreuther, Erik H. J. Danen and Nils Cordes

Supplementary Figures

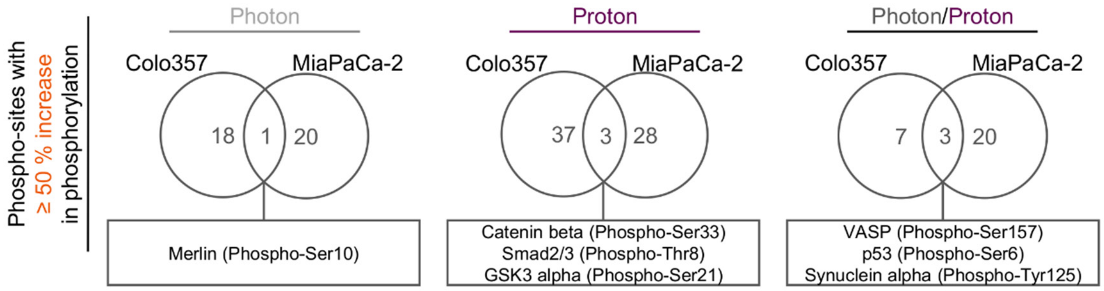

Figure S1. Changes in the phosphoproteome of 3D PDAC cell cultures by proton and photon irradiation. Venn diagram analysis comparing phospho-sites altered by ≥ 50 % increase in phosphorylation of Colo357 and MiaPaCa-2 cell lines either specifically after photons and protons or by both kinds of irradiation.

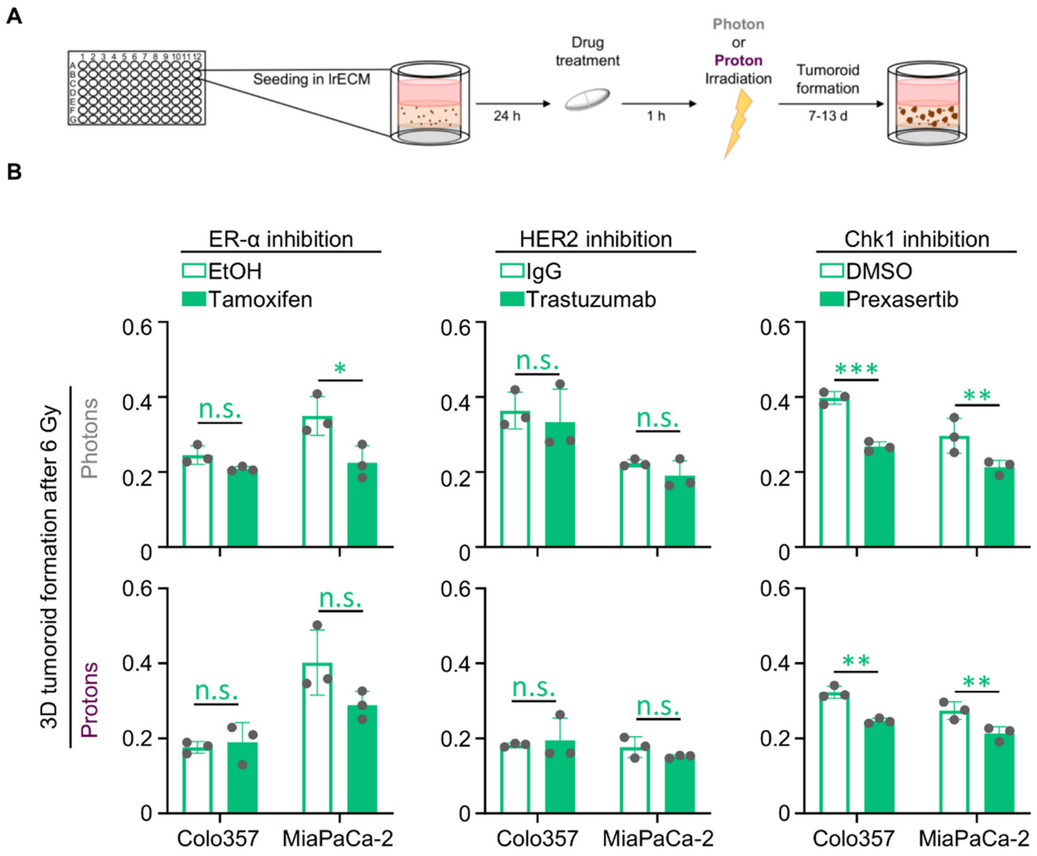

Figure S2. Photon and proton irradiated 3D PDAC cell cultures differentially respond to tamoxifen, trastuzumab and prexasertib. (**A**) Experimental set-up for examining 3D PDAC tumoroid growth; (**B**) 3D tumoroid formation capacity upon 1-h pretreatment with tamoxifen, trastuzumab or prexasertib in combination with 6-Gy photon or proton irradiation. All results show mean ± SD (n = 3; two-sided t test; *, $P < 0.05$; **, $P < 0.01$; ***, $P < 0.001$).

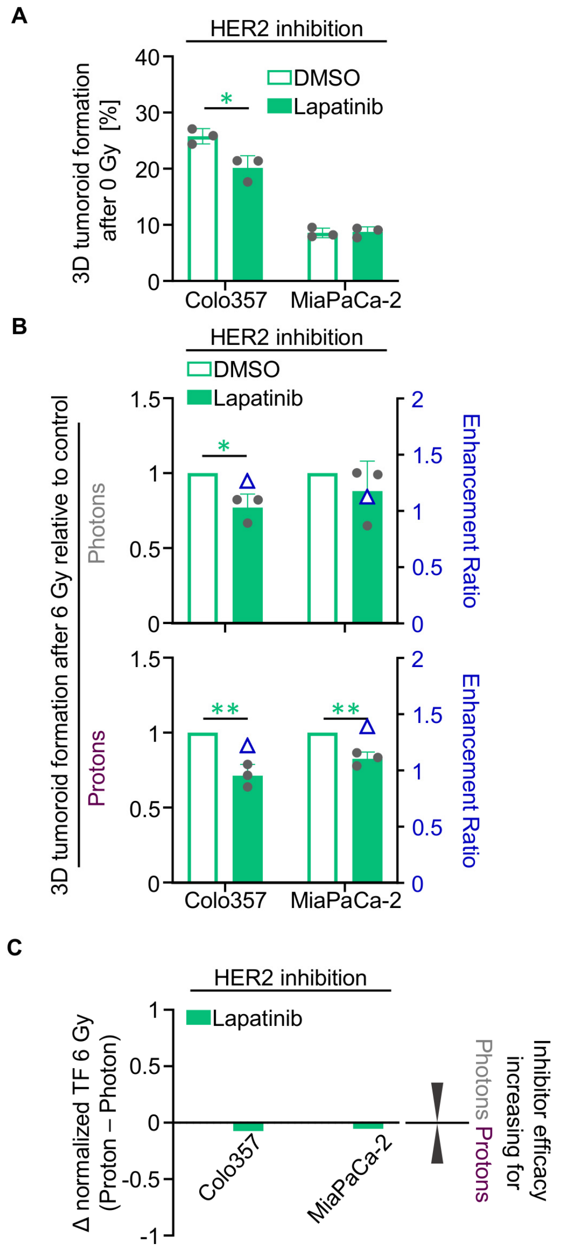

Figure S3. Photon and proton irradiated 3D PDAC cell cultures differentially respond to lapatinib. (**A**) 3D tumoroid formation capacity of unirradiated Colo357 and MiaPaCa-2 cells treated with lapatinib (experimental set-up shown in Fig. S2A); (**B**) Normalized 3D tumoroid formation capacity upon 1-h pretreatment with lapatinib in combination with 6-Gy photon or proton irradiation. Enhancement ratio (tumoroid formation capacity after 6 Gy control treatment/tumoroid formation capacity after 6 Gy inhibitor treatment) are indicated as blue triangles; (**C**) Differences in the radiosensitizing efficacy of inhibitors visualized by Δ values of normalized tumor formation capacity (tumoroid formation capacity after 6 Gy of protons - tumoroid formation capacity after 6 Gy of photons). All results show mean ± SD (n = 3; two-sided t test; *, $P < 0.05$; **, $P < 0.01$); Δ: delta, TF: tumoroid formation.

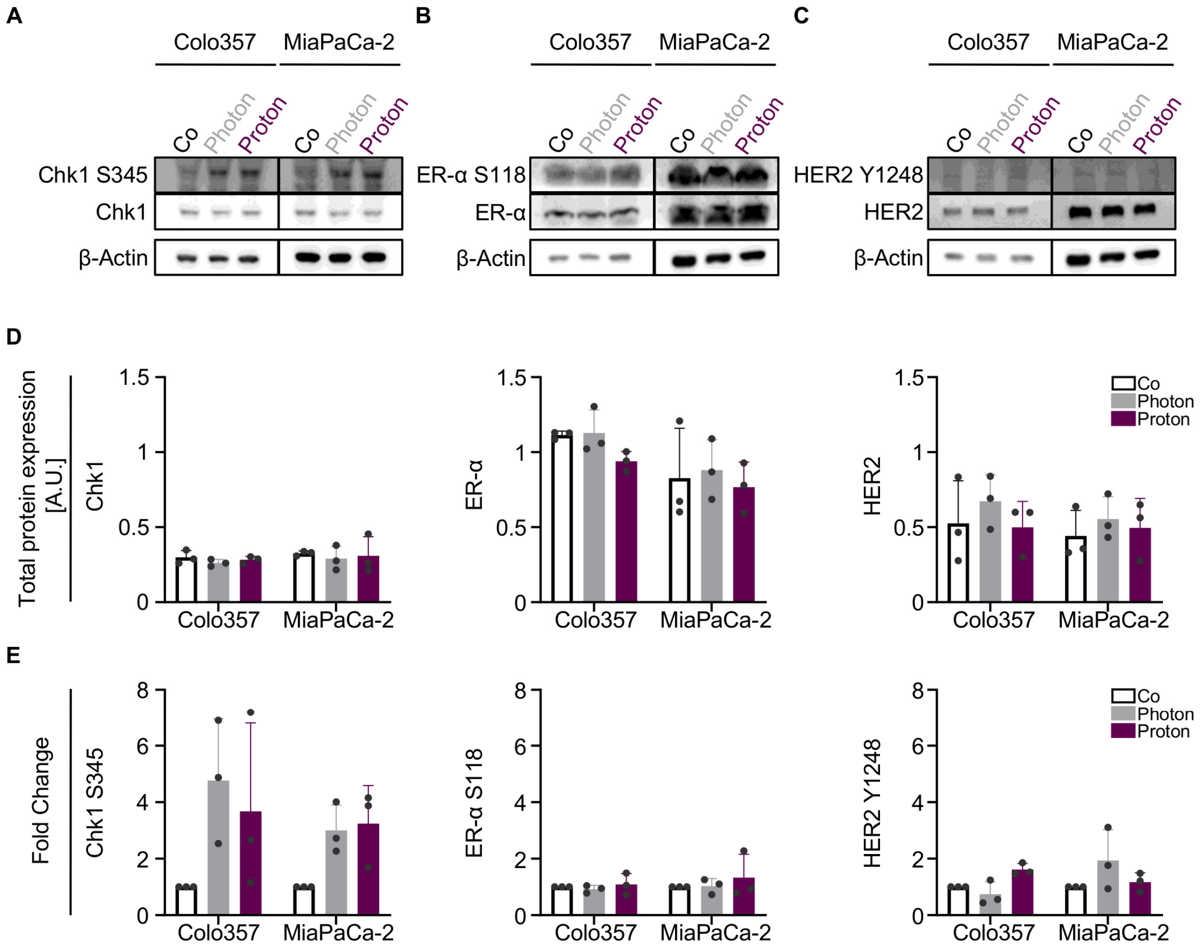

Figure S4. Photon and proton irradiation differentially affect the phosphorylation status of Chk1, ER-α and HER2 of 3D PDAC cell cultures. (**A, B, C**) Immunoblots on whole cell lysates from 0-Gy (Co) or 6-Gy photon or 6-Gy proton irradiated Colo357 and MiaPaCa-2 cells showing total and phosphorylated forms of Chk1, ER-α and HER2. β-Actin served as loading control. (**D**) Densitometry of normalized total forms of Chk1, ER-α and HER2 from A, B and C. (**E**) Fold change of phosphorylated forms of Chk1, ER-α and HER2 from A, B and C. All results show mean ± SD (n = 3); A.U.: arbitrary unit, Co: control.

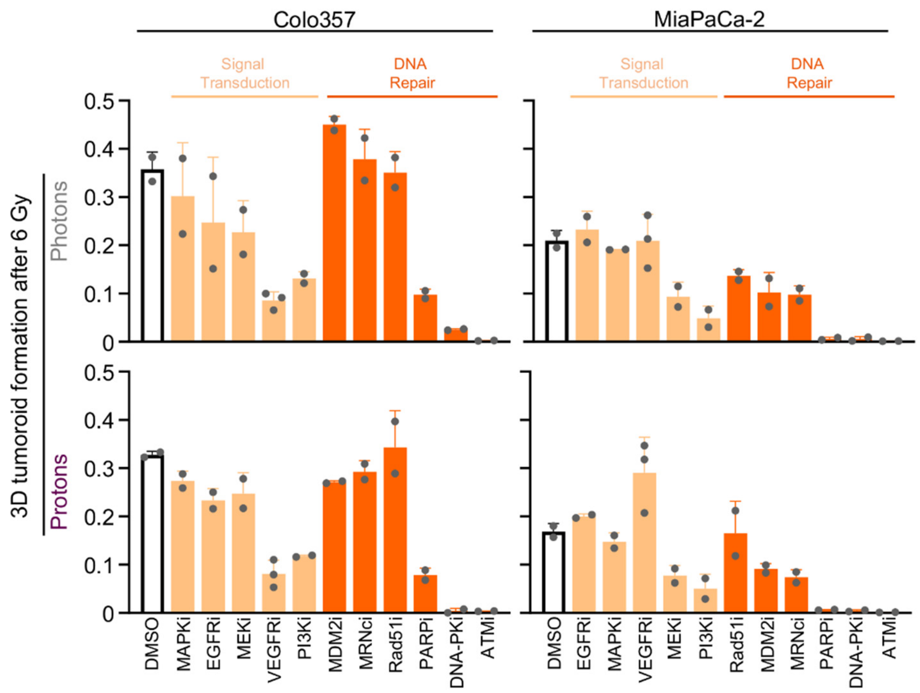

Figure S5. Targeting of signal transduction and DNA repair enzymes in sensitizes PDAC cell cultures to photon and proton irradiation. 3D tumoroid formation capacity upon 1-h inhibitor pretreatment combined with 6-Gy photon or proton irradiation. All results show mean ± SD (n ≥ 2).

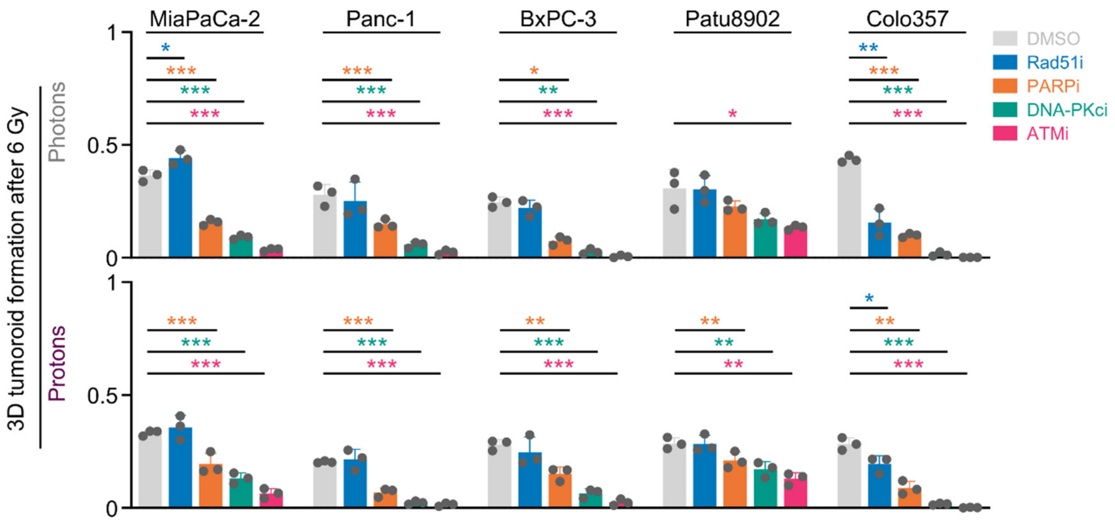

Figure S6. Targeting NHEJ associated enzymes seems generally potent to sensitize 3D PDAC cell cultures to photon and proton irradiation. 3D tumoroid formation capacity upon 1-h inhibitor pretreatment combined with 6-Gy photon or proton irradiation. All results show mean ± SD (n = 3; two-sided t test; *, $P < 0.05$; **, $P < 0.01$; ***, $P < 0.001$).

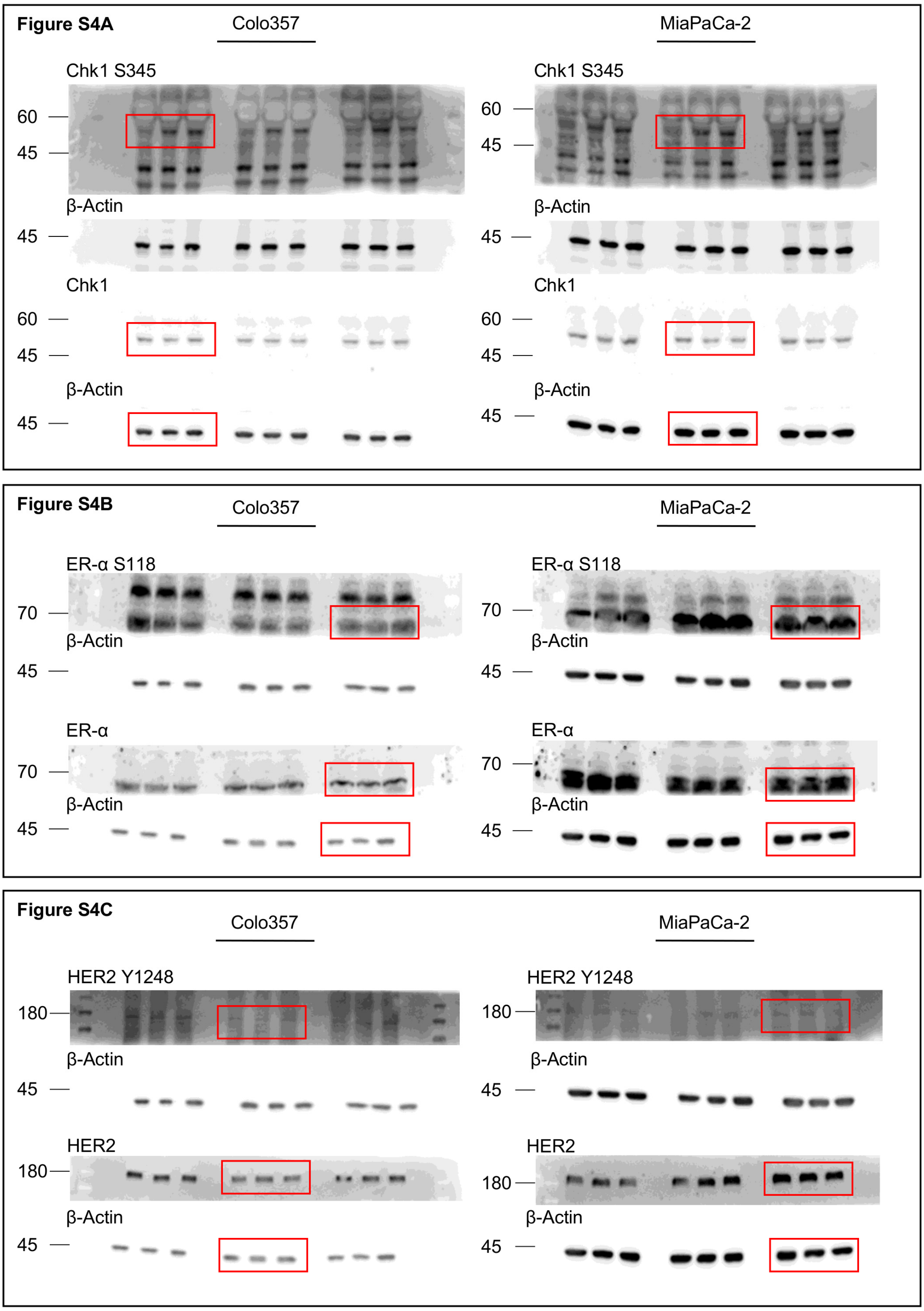

Figure S7. Compilation of uncropped immunoblots for Figure S4A-C. The corresponding main figures where the cropped versions are shown are specified on top of the blots. The red rectangles display the areas of the plots that were used in Figure S4A-C. The molecular weight standard [kDa] per blot is located on the left. The detected protein is indicated on top of the blots.

Supplementary Tables

Table S1. RBE values comparing proton to photon irradiation effectiveness in PDAC cell lines.

Cell Line	RBE
MiaPaCa-2	1.2
Panc-1	1.7
BxPC-3	0.6
Patu8902	1.4
Colo357	2.1

Table S2. SF2 values of PDAC cell lines after photon and proton irradiation.

Cell Line	SF2 Gy Photon	SF2 Gy Proton
MiaPaCa-2	0.69	0.61
Panc-1	0.58	0.34
BxPC-3	0.56	0.61
Patu8902	0.70	0.51
Colo357	0.73	0.51

Table S3. Changes in the phosphoproteome of 3D Colo357 cell cultures upon proton or photon irradiation. Alterations of phosphorylation sites in Colo357 cells upon 6 Gy-photon or -proton irradiation after normalization to 0 Gy; data in the order of fold change (high to low) of photon induced changes.

Phospho-site	Photon	Proton
Ret (Phospho-Tyr905)	2.70063991	2.17900741
c-Jun (Phospho-Thr93)	2.45253332	2.98009779
Raf1 (Phospho-Ser259)	2.39273888	1.52443335
Progesterone Receptor (Phospho-Ser190)	2.1791565	1.09130718
Myc (Phospho-Thr358)	2.1556917	2.06737326
Smad1 (Phospho-Ser187)	1.98893291	1.15605866
CaMK2A (Phospho-Thr286)	1.98326727	1.00479118
p38 MAPK (Phospho-Tyr182)	1.9786095	1.42266531
JAK2 (Phospho-Tyr221)	1.84789351	1.14415143
B-RAF (Phospho-Thr598)	1.76563059	1.20751251
JunB (Phospho-Ser79)	1.71460595	0.94995046
Chk1 (Phospho-Ser317)	1.70814535	1.1799637
Merlin (Phospho-Ser10)	1.70567351	0.74910004
VEGFR2 (Phospho-Tyr1214)	1.70482966	1.19974658
SHP-2 (Phospho-Tyr542)	1.70311842	1.91802862
ATF2 (Phospho-Thr69/51)	1.69764565	1.43928945
Synuclein alpha (Phospho-Tyr125)	1.63698527	1.53090582
p53 (Phospho-Ser6)	1.62937698	2.41819121
VASP (Phospho-Ser157)	1.5991608	1.54943129
PKC delta (Phospho-Ser645)	1.59735986	1.89258878
Rel (Phospho-Ser503)	1.58767633	0.75961686
BLNK (Phospho-Tyr96)	1.58540286	1.3699548

BCR (Phospho-Tyr177)	1.56788116	1.34324107
Myc (Phospho-Ser62)	1.5524829	1.51849616
KIT (Phospho-Tyr936)	1.55060283	1.01841641
P90RSK (Phospho-Thr359/Ser363)	1.54011285	1.48606198
HSP27 (Phospho-Ser82)	1.52795653	1.14581854
ICAM-1 (Phospho-Tyr512)	1.5265309	1.39545342
Estrogen Receptor-alpha (Phospho-Ser167)	1.50857962	1.20830175
MEK1 (Phospho-Thr291)	1.48228625	1.90442439
GABA-RB (Phospho-Ser434)	1.4054177	1.50425061
FKHR (Phospho-Ser256)	1.34986614	1.50049394
HDAC8 (Phospho-Ser39)	1.31556498	2.05665571
AMPK1/AMPK2 (Phospho-Ser485/491)	1.31431106	1.59200518
Shc (Phospho-Tyr349)	1.30686317	1.81838205
LKB1 (Phospho-Thr189)	1.29338126	1.66974721
MARCKS (Phospho-Ser163)	1.24701261	1.60057644
LIMK1 (Phospho-Thr508)	1.23044136	0.64018335
IR (Phospho-Tyr1361)	1.20204333	1.60053413
STAT6 (Phospho-Thr645)	1.19342586	1.53553826
NFkB-p65 (Phospho-Ser276)	1.19253845	1.82927185
Rac1/cdc42 (Phospho-Ser71)	1.18113105	1.7964397
FAK (Phospho-Tyr861)	1.17525367	1.5247009
Ras-GRF1 (Phospho-Ser916)	1.15163461	0.62467844
SYK (Phospho-Tyr525)	1.1490482	0.65631925
Integrin beta-3 (Phospho-Tyr785)	1.1398226	1.90470634
IL-4R/CD124 (Phospho-Tyr497)	1.13786696	1.55179048
SAPK/JNK (Phospho-Tyr185)	1.13158284	1.52777734
c-Jun (Phospho-Ser243)	1.13090858	1.76043884
Raf1 (Phospho-Ser43)	1.11995903	0.59497579
ATF4 (Phospho-Ser245)	1.0980444	2.09308654
AKT1 (Phospho-Tyr474)	1.09640995	1.51336031
Histone H2A.X (Phospho-Ser139)	1.09261964	0.5888499
EGFR (Phospho-Tyr1069)	1.09218295	1.78614458
JunD (Phospho-Ser255)	1.07129202	1.65802969
Src (Phospho-Tyr529)	1.06801471	1.88238771
IRS-1 (Phospho-Ser636)	1.06455254	1.63591427
HNF4 alpha (Phospho-Ser313)	1.05989507	1.52074853
Zap-70 (Phospho-Tyr319)	1.05497803	1.50537894
Catenin beta (Phospho-Ser33)	1.05326336	1.62758358
GSK3 alpha (Phospho-Ser21)	1.04457457	2.05284178
HER2 (Phospho-Tyr1248)	1.03852483	1.67173687
MEK1 (Phospho-Ser221)	1.03082342	0.62305104
Stathmin 1 (Phospho-Ser37)	1.0219395	1.53928559
GluR1 (Phospho-Ser863)	1.02161512	1.53252141
PKC zeta (Phospho-Thr410)	1.01841503	0.6454938
Src (Phospho-Tyr418)	0.99741722	0.6674143
Tau (Phospho-Ser235)	0.99690644	0.63050923
HSP90B (Phospho-Ser254)	0.9917134	0.60779095
p27Kip1 (Phospho-Thr187)	0.98348729	1.69426297
PAK3 (Phospho-Ser154)	0.9684073	1.58266132
p53 (Phospho-Ser15)	0.96313904	0.68580232
Kv1.3/KCNA3 (Phospho-Tyr135)	0.95782663	1.65050849
p53 (Phospho-Thr18)	0.95361538	1.52339744

P70S6K (Phospho-Ser424)	0.93712208	1.58327759
EGFR (Phospho-Thr678)	0.9188876	0.64955752
SP1 (Phospho-Thr739)	0.88683661	0.62651589
Smad2/3 (Phospho-Thr8)	0.88243607	1.60691098
Integrin beta-4 (Phospho-Tyr1510)	0.86915265	0.68943398
HSP90B (Phospho-Ser226)	0.86887939	1.99080699
Catenin beta (Phospho-Thr41/Ser45)	0.8525006	0.682244
Cyclin D1 (Phospho-Thr286)	0.82098739	1.58053313
EEF2 (Phospho-Thr56)	0.81276026	0.60643045
NFkB-p65 (Phospho-Thr435)	0.78788937	1.70770431
ETK (Phospho-Tyr40)	0.77033365	0.62065896
Abl1 (Phospho-Tyr204)	0.76143802	0.43925468
STAT4 (Phospho-Tyr693)	0.76021291	0.68216386
GluR1 (Phospho-Ser849)	0.74596352	0.39675663
Synaptotagmin (Phospho-Thr202)	0.74484621	0.67370037
BRCA1 (Phospho-Ser1457)	0.73801274	0.62182356
BTK (Phospho-Tyr223)	0.7328498	0.58613631
c-Jun (Phospho-Ser63)	0.69651036	1.28411481
CDK2 (Phospho-Thr160)	0.69059098	0.91193142
MSK1 (Phospho-Thr581)	0.67466541	0.97537095
IKK-alpha/beta (Phospho-Ser180/181)	0.66848208	0.57919427
DAPP1 (Phospho-Tyr139)	0.66347416	0.5308503
Tau (Phospho-Thr212)	0.60429967	2.26777101
JunB (Phospho-Ser259)	0.58304899	0.99686023
EGFR (Phospho-Tyr1110)	0.55972525	1.2079582
IRS-1 (Phospho-Ser312)	0.55379573	0.89430778
CDC25C (Phospho-Ser216)	0.49143091	0.3439015
Cortactin (Phospho-Tyr421)	0.4224753	0.46944096
Elk1 (Phospho-Thr417)	0.41500116	1.11929826
VEGFR2 (Phospho-Tyr1175)	0.37927802	0.42769493
HDAC1 (Phospho-Ser421)	0.37347245	0.37562516

Table S4. Changes in the phosphoproteome of 3D MiaPaCa-2 cell cultures upon proton or photon irradiation. Alterations of phosphorylation sites in MiaPaCa-2 cells upon 6 Gy-photon or -proton irradiation after normalization to 0 Gy; data in the order of fold change (high to low) of photon induced changes.

Phospho-site	Photon	Proton
GluR1 (Phospho-Ser849)	3.12639767	1.62091688
Chk1 (Phospho-Ser317)	2.85340647	3.96590262
IRS-1 (Phospho-Ser312)	2.45175548	1.60138521
Chk1 (Phospho-Ser286)	2.43553012	1.28299702
c-Jun (Phospho-Ser243)	2.31396824	1.35734707
Cortactin (Phospho-Tyr421)	2.29826829	1.12561208
4E-BP1 (Phospho-Thr45)	2.26997323	2.30210331
p44/42 MAPK (Phospho-Thr202)	2.17424821	1.59633561
CREB (Phospho-Ser133)	2.09651235	1.38341264
GAP43 (Phospho-Ser41)	2.04650163	1.54533559
Merlin (Phospho-Ser10)	1.99347151	1.24975263
EGFR (Phospho-Tyr1110)	1.93990268	1.99450638
HDAC8 (Phospho-Ser39)	1.93672295	1.64121421

LCK (Phospho-Tyr393)	1.88731204	1.85101485
PDGFR beta (Phospho-Tyr751)	1.84659234	1.54870689
NFkB-p105/p50 (Phospho-Ser337)	1.79599404	2.07168904
PPAR-gamma (Phospho-Ser112)	1.7929958	2.16171167
Synuclein alpha (Phospho-Tyr125)	1.78866058	1.55638597
IR (Phospho-Tyr1361)	1.78386459	1.48031653
STAT5A (Phospho-Ser780)	1.76989368	1.10563231
14-3-3 theta/tau (Phospho-Ser232)	1.7008973	1.69523956
p53 (Phospho-Ser6)	1.67979144	1.65533886
4E-BP1 (Phospho-Thr36)	1.6274711	1.27145006
HSP27 (Phospho-Ser82)	1.62004044	1.50622336
NFkB-p65 (Phospho-Ser529)	1.61637705	1.70177006
VASP (Phospho-Ser157)	1.61195941	1.73878373
Tau (Phospho-Ser396)	1.60548821	1.09918416
p44/42 MAPK (Phospho-Tyr204)	1.59901573	1.070279
IGF1R (Phospho-Tyr1165/1166)	1.59104721	1.44576845
Gab1 (Phospho-Tyr627)	1.58918178	1.84014816
VAV1 (Phospho-Tyr174)	1.58671754	0.77458437
GSK3 beta (Phospho-Ser9)	1.57531557	1.62847302
P70S6K-beta (Phospho-Ser423)	1.56506454	1.42650605
E2F1 (Phospho-Thr433)	1.56034127	1.33098781
BTK (Phospho-Tyr223)	1.55613193	1.00922394
STAT6 (Phospho-Tyr641)	1.54838595	1.43354543
Integrin beta-3 (Phospho-Tyr773)	1.54085619	1.43081457
BCL-XL (Phospho-Thr47)	1.53771072	1.31320501
Tau (Phospho-Thr205)	1.53268363	0.85844836
Tau (Phospho-Thr212)	1.53215599	3.24712749
HER4/ErbB4 (Phospho-Tyr1284)	1.52065808	1.69724387
mTOR (Phospho-Thr2446)	1.52032579	1.4698677
Estrogen Receptor-alpha (Phospho-Ser118)	1.50590146	1.20915191
DAPP1 (Phospho-Tyr139)	1.50133929	1.56357363
Lamin A (Phospho-Ser22)	1.49914622	1.73000303
Smad2/3 (Phospho-Thr8)	1.49687989	1.65054288
BCR (Phospho-Tyr360)	1.46675016	1.51033759
Ezrin (Phospho-Tyr353)	1.45843251	2.47356486
STAM2 (Phospho-Tyr192)	1.44297722	1.56476404
A-RAF (Phospho-Tyr301/302)	1.43130081	1.52488069
PDK1 (Phospho-Ser241)	1.41883428	1.58353023
Vinculin (Phospho-Tyr821)	1.39679387	1.81339106
Catenin delta-1 (Phospho-Tyr228)	1.3959914	1.58560181
Tau (Phospho-Thr181)	1.38963801	1.68892844
PLD1 (Phospho-Ser561)	1.38916409	1.51103466
c-Jun (Phospho-Tyr170)	1.37760676	2.45252605
HER2 (Phospho-Thr686)	1.37560713	1.68209936
MEK1 (Phospho-Ser221)	1.33066999	1.88869289
VASP (Phospho-Ser238)	1.3000635	2.07349381
IkB-alpha (Phospho-Ser32/36)	1.28742415	1.65769549
HDAC4 (Phospho-Ser632)	1.28300746	1.52700163
M-CSF Receptor (Phospho-Tyr561)	1.28285249	1.61861654
NMDAR1 (Phospho-Ser897)	1.26685881	1.5113497
Paxillin (Phospho-Tyr31)	1.19412301	1.64788675
DARPP-32 (Phospho-Thr34)	1.18431612	0.47569792

Ret (Phospho-Tyr905)	1.18030293	1.64208642
Caspase 9 (Phospho-Ser196)	1.16781639	1.54915257
Pyk2 (Phospho-Tyr402)	1.14672085	2.00770347
Catenin beta (Phospho-Ser33)	1.13318021	1.5973677
MEF2A (Phospho-Thr312)	1.13006756	0.63855859
HSP27 (Phospho-Ser78)	1.11363636	1.55764463
Smad3 (Phospho-Ser213)	1.06740448	1.60195119
Estrogen Receptor-alpha (Phospho-Ser104)	1.05165122	0.6765868
CrkII (Phospho-Tyr221)	1.04814795	0.64896327
Elk1 (Phospho-Thr417)	1.01085653	1.50043422
VEGFR2 (Phospho-Tyr951)	0.9616111	2.07199518
Caspase 9 (Phospho-Tyr153)	0.93238488	0.69321767
eNOS (Phospho-Ser1177)	0.90781166	0.65443808
HDAC1 (Phospho-Ser421)	0.90420502	0.43203522
Smad3 (Phospho-Ser425)	0.89278843	1.57594248
AKT1S1 (Phospho-Thr246)	0.87937691	0.59376814
SP1 (Phospho-Thr739)	0.87765257	1.85070949
NFkB-p100/p52 (Phospho-Ser869)	0.85522206	1.51664396
PKC delta (Phospho-Ser645)	0.84378043	0.63649156
HER2 (Phospho-Tyr1221/Tyr1222)	0.83719472	0.65992535
GSK3 alpha (Phospho-Ser21)	0.80033461	2.56288739
Synaptotagmin (Phospho-Thr202)	0.79481595	0.61711692
MKK4/SEK1 (Phospho-Thr261)	0.76834902	0.60062515
IKK-gamma (Phospho-Ser31)	0.76325188	0.61388936
MAP3K8/COT (Phospho-Thr290)	0.75770293	0.6939146
EGFR (Phospho-Tyr1172)	0.74237332	0.48313306
Estrogen Receptor-alpha (Phospho-Ser106)	0.73979133	0.6802518
SHP-2 (Phospho-Tyr580)	0.73568223	0.63298831
NFkB-p105/p50 (Phospho-Ser927)	0.69390873	0.54647309
LYN (Phospho-Tyr507)	0.67747666	0.5419336
ATP1A1/Na+K+ ATPase1 (Phospho-Ser23)	0.67328506	1.03358422
STAT4 (Phospho-Tyr693)	0.67151974	1.00142745
FAK (Phospho-Tyr397)	0.67114155	0.69886877
Rel (Phospho-Ser503)	0.67093154	0.95819247
Ras-GRF1 (Phospho-Ser916)	0.66848779	0.76341485
Shc (Phospho-Tyr349)	0.64535247	0.82144882
FAS (Phospho-Tyr291)	0.61267418	0.80353981
GluR2 (Phospho-Ser880)	0.60318531	0.68940892
Rb (Phospho-Ser780)	0.58182929	0.49950186
Abl1 (Phospho-Tyr204)	0.52726965	0.51344122
4E-BP1 (Phospho-Ser65)	0.51014307	0.5576899
BRCA1 (Phospho-Ser1457)	0.51001129	0.83262298
SYK (Phospho-Tyr525)	0.49291706	0.53658079
Raf1 (Phospho-Ser296)	0.45110474	0.69255234
Cyclin D1 (Phospho-Thr286)	0.39552343	0.57634133
